# Modification of the active centre of nattokinase to enhance its thermostability using a strategy based on molecular dynamics simulation, steered dynamics simulation, and conservative prediction

**DOI:** 10.3389/fnut.2024.1505584

**Published:** 2024-11-14

**Authors:** Yuan Li, Wenhui Zhu, Liangqi Chen, Xiyu Tang, Aixia Ma, Yuwei Ma, Tongli Li, Xingrui Li, Ye Ma, Jinyao Li

**Affiliations:** ^1^Xinjiang Key Laboratory of Biological Resources and Genetic Engineering, College of Life Science and Technology, Xinjiang University, Urumqi, China; ^2^School of Pharmaceutical Sciences and Institute of Materia Medica, Xinjiang University, Urumqi, China

**Keywords:** nattokinase, thermostability, active center region, rational design, molecular dynamics

## Abstract

**Introduction:**

The poor thermostability of nattokinase represents a significant limitation in its potential applications. Additionally, there is a notable absence of studies focused on modifying residues within the active site region of nattokinase with the aim of enhancing its catalytic properties. Furthermore, the direct utilisation of directed evolution often yields unfavourable outcomes, with a considerable workload being a common consequence.

**Methods:**

In order to solve the above problems, a new method based on molecular dynamics simulation, steered dynamics simulation and conservative analysis with site-directed mutagenesis was proposed to screen nattokinase mutants with improved thermal stability. Molecular dynamics simulation was used to explain the mechanism of catalytic performance improvement of positive mutants. Finally, the fermentation process of the positive mutant was optimized.

**Results and discussion:**

Based on these findings, the mutant A216K was selected for a 5.7-fold increase in half-life at 55°C with a small increase in activity, which further enhanced the mutation library of the thermal stability enhancement site in the enzyme’s active centre. The results of the molecular dynamics simulation indicated that the enhancement of the number of hydrogen bonds within the protein and between the protein and the solvent, as well as the augmentation of the rigidity around the calcium ion binding site and the mutation site, were the primary factors contributing to the improvement of the thermal stability of A216K. It is anticipated that this strategy will provide novel insights into enzyme engineering research.

## 1 Introduction

Nattokinase is a potent fibrinolytic enzyme from *Bacillus subtilis* var. natto, found in the traditional soy food natto. It has been reported to directly digest fibrin, especially its cross-linked form (1, 2). Unlike proteins with thrombolytic activity currently used in the health food and pharmaceutical markets, nattokinase presents itself as a valuable alternative due to its low bleeding risk, high tolerance dose, and lack of gene mutation or induced chromosome aberration (3, 4). It has multiple thrombolytic mechanisms, including hydrolyzing thrombus into amino acids and small peptides, activating prourokinase, and promoting tissue plasminogen activator production (1, 5). The nattokinase gene, part of the subtilis protease family, is 381 amino acids long (6). Genetically engineered bacterial strains, such as *Escherichia coli* (7, 8), *Lactobacillus* (9), *Bacillus* (10), and *Komagataella phaffii* (11), have successfully expressed nattokinase. Its 3D structure includes nine α-helices and nine β-folds, with two Ca^2+^ binding sites for stability (12). The catalytic activity centre of nattokinase comprises conserved catalytic triplets (Asp_32_, His_64_, and Ser_221_), while the substrate binding centre is made up of three conserved amino acids (Ser_125_, Leu_126,_ and Gly_127_) (13).

The poor thermostability of nattokinase limits its industrial production and market application, making its enhancement a key research focus (14). Compared to traditional chemical modification, gene editing for molecular modification of enzyme molecules offers several advantages: it enhances the stability of the enzymes, effectively lowers production costs, and is more energy-efficient and environmentally friendly. As a result, it has emerged as a leading method in enzyme engineering research (15). Enzyme modification methods typically involve directed evolution or rational design. Rational design is a protein modification strategy that relies on the known relationship between amino acid sequence, protein structure, and function (16). The current application of enzyme modification methods to improve the thermal stability of nattokinase is the use of computer-aided algorithms and flexible region design (17–19).

The catalytic centre region of the enzyme constitutes the core region responsible for the enzyme’s catalytic function. Consequently, modifying this region can enhance the enzyme’s catalytic performance to a greater extent than modifying other regions (20). The current thermostability modification strategies for nattokinase do not involve modification of the catalytic centre. However, some reported thermostability-enhancing nattokinase mutants are in fact located in the catalytic centre region. This includes mutants such as S33T, S62A, Y217K, and N218L, which confirm the important influence of the active centre of nattokinase on its thermostability (17, 18, 21). Modifying the active centre of an enzyme is a more challenging process than modifying other regions. This is due to the fact that the amino acid residues in the active centre of an enzyme are highly conserved and interact extensively with the substrate. This makes it more likely that the enzyme will become inactivated following a mutation in this region. Additionally, the active centre of an enzyme has a high number of sites, which increases the workload associated with saturation mutation. It is therefore evident that a rational design strategy to enhance the thermostability of the enzyme by modifying its active centre represents an effective means of solving the aforementioned problems.

In summary, in order to systematically explore the sites in the active center of nattokinase that affect the thermostability of the enzyme while improving the prediction accuracy, a new strategy based on the combination of molecular dynamics simulation at different temperatures, steered molecular dynamics simulation, and sequence conservative analysis (MSC) was designed in this study. The initial identification of candidate sites with high flexibility changes was achieved through a comparative analysis of RMSF alterations in the active centre of nattokinase under varying temperature conditions. This was followed by the utilisation of steered molecular dynamics simulation to eliminate sites involved in substrate interactions upon substrate entry into the enzyme’s active centre. Subsequently, the highly conserved sites were further excluded through a sequence conservative analysis. By comparing the results with those obtained from the traditional directed evolution of amino acid residues in the active centre region, this method improves the efficiency of the screening process while addressing the challenge of the activity-stability trade-off in enzyme engineering research. Furthermore, it offers novel insights and solutions for the modification of enzyme active centres.

## 2 Materials and methods

### 2.1 Plasmids, strains, and cultivation conditions

The recombinant plasmid pET-28a-*AprY* (resistant to kanamycin) was used as the expression vector, which contained a full nattokinase peptide (signal peptide + propeptide + mature peptide, recombinant AprY, rAprY) from *Bacillus mojavensis* LY-06 (7). *Escherichia coli* BL21 (DE3; *E. coli* BL21) was used as the expression host for rAprY. Plasmid propagation and overexpression were carried out using Luria-Bertani (LB) medium containing 5 g/L yeast extract, 10 g/L tryptone, and 10 g/L NaCl at pH 7.0. Recombinant protein expression was induced using Isopropyl-beta-D thiogalactopyranoside (IPTG).

### 2.2 Bioinformatics analysis

The protein sequences of rAprY and its mutants were submitted to the Alfafold workstation, which generated accurate structural models (https://alphafoldserver.com/, accessed on 11 January 2024) (22). The protein models were then analysed using the PROCHECK module of SAVES 6.0 to generate a Ramachandran plot and assess the model’s reliability (Supplementary Figure S1) (23). The conservation of the AprY primary sequence was calculated using Consurf-DB online software (https://consurf.tau.ac.il/consurf_index.php, accessed on 11 January 2024) (24). Docking was performed using AutoDock using the default docking parameters supplied with AutoDock in the ‘examples’ subdirectory, and point charges initially assigned according to the AMBER03 force field, and then damped to mimic the less polar Gasteiger charges used to optimize the AutoDock scoring function (25).

The protein models underwent molecular dynamic simulation (MD) using YASARA (Yet Another Scientific Artificial Reality Application) software (26). The hydrogen-bonding network (27) was optimized to enhance solute stability. Additionally, a pKa prediction was performed to adjust the protonation states of protein residues at pH 8. NaCl were added to achieve a physiological concentration of 0.9%, with an excess of either Na or Cl to neutralize the cell. After performing steepest-descent and simulated-annealing minimizations to eliminate clashes, the simulation was conducted for 100 nanoseconds using the AMBER14 force field for the solute and TIP3P for water (28). Van der Waals forces were calculated using a cutoff of 10 angstroms. Long-range electrostatics were calculated using the particle-mesh Ewald method (29) with a distance of 1.0 nanometer. The equations of motion were integrated using algorithms that have been previously described in detail (30). Bonded and non-bonded interactions were calculated using different multiple timesteps of 1.25 and 2.5 fs, respectively. The simulation was conducted in the NPT ensemble at a temperature of 328.15 K and a pressure of 1 atm. The first 50 ns were excluded from further analysis as they were considered equilibration time, based on inspection of the solute RMSD over time. For this analysis, we selected several programs, including macro md_run for running the simulation, md_analyze to analyze the radius of gyration (Rg), potential energy, solvent accessible surface area (SASA), protein secondary structure content, and number of hydrogen bonds, and md_analyzeres to obtain the root mean square fluctuation (RMSF) value. We visualized the enzyme structure using Pymol software.

Steered molecular dynamics simulation (SMD), which is used to illustrate how biomolecules respond to external mechanical manipulations at the atomic level, mimics the principles of atomic force microscopy (AFM) (31). The equations of motion were integrated using a multiple timestep of 1.25 fs for bonded intraactions and 2.5 fs for non-bonded interactions at a temperature of 298 K and a pressure of 1 atm (NPT ensemble) using algorithms previously described in detail (30). After a 3 ps equilibration period, a minimum acceleration of 2,000 pm/ps^2^ was applied to all ligand atoms, along with non-bonded forces every 2.5 fs. The ligand has a mass of 624.24 Daltons, and using the equation F = m*a, this results in a pulling force of [2000*624.24*0.00166] picoNewtons. The pulling direction was determined as the vector connecting the centers of mass of the receptor and ligand, and was continuously updated to account for rotations of the complex. The simulation continuously updated the maximum distance between the centers of mass of the receptor and ligand. If the distance did not increase for 400 simulation steps, the acceleration was increased by 500 pm/ps^2^. When the maximum distance grew with a ‘MaxDisSpeed’ faster than 4,000 m/s, the acceleration was scaled down by a factor of 1−(1−4000/MaxDisSpeed)^2^, but not below the initial minimum. This check occurred every 20 simulation steps. The simulation ended when the ligand had moved 15 Å away. The peak pulling force and total work done were calculated to correlate with the binding strength.

### 2.3 Bacterial expression and purification of rAprY and its mutants

The method for expressing and purifying rAprY and its mutants is based on the previous research conducted by Li et al. (17). *Escherichia coli* BL21 (DE3) was used as the host for protein induction, and overexpression was achieved after 20 h of incubation at 18°C by adding 0.1 mM IPTG. Following induction, the cells were collected by centrifugation and sonicated. The supernatant (crude enzyme) obtained by the centrifugation of cell lysates was subjected to Ni-NTA column (Invitrogene, Carlsbad, CA, USA) and DEAE Sepharose Fast Flow column (Amersham Biosciences, Piscataway, NJ, USA) for protein purification. The protein concentration was determined using the BCA protein assay reagent kit (Pierce).

### 2.4 Enzymatic activity assays

Fibrin plate assay for primary screening of crude enzyme activity and stability at nattokinase candidate sites and validation of catalytic properties of A216E, A216K, and A216R pure enzymes. A solution of 4 mg/ml fibrinogen (prepared in a water bath at 37°C; Shanghai yuanye Bio-Technology Co., Ltd), 50 U/ml thrombin (Shanghai yuanye Bio-Technology Co., Ltd), and 1% agarose (prepared in a water bath at 60°C) was prepared using a sodium barbital buffer (comprising 10.1 g/L barbital sodium, 7.4 g/L NaCl, 1 g/L gelatin, and a pH of 7.8). A total of 7.5 ml of the 1% agarose solution was combined with 7.5 ml of the 4 mg/ml fibrinogen solution and 0.4 ml of the 50 U/ml thrombin solution. The resulting mixture was then poured into the plate and left at room temperature for 30 min. A total of 1 μl of the nattokinase crude enzyme was added to the aforementioned plate, and the diameter of the transparent circle (mm^2^) was calculated after culturing at 37°C for 18 h. The fibrinolytic activity was then measured using a urokinase standard as a control (32).

The kinetic parameters were measured by means of the chromogenic substrate N-succinyl-L-Ala-L-Ala-L-Pro-L-Phe-p-nitroanilide (sucAAPF-pNA), as described previously (33). The kinetic parameters of rAprY and its mutants were determined using sucAAPF-pNA at concentrations ranging from 0.05 to 5 mM in PBS buffer (Na_2_HPO_4_ 8 mM, NaCl 136 mM, KH_2_PO_4_ 2 mM, KCl 2.6 mM, pH 7.0–7.2) at 25°C. The *K*_m_ and *k*_cat_ values were calculated using the Michaelis–Menten method. GraphPad Prism 8 software (San Diego, CA) was used for this purpose. The presented values are the means of three independent experiments (33). One unit was defined as the amount of enzyme that produced 1 μmol p-NA per minute under assay conditions.

### 2.5 Biochemical characterization of rAprY and its mutants

The tetrapeptide substrate method was used for the characterisation of enzymatic properties and determination of kinetic parameters of A216E, A216K, A216R, and P225A pure enzymes. Determination of the optimal temperature: the pure enzyme solution was placed in different temperature conditions (50–61°C), and the enzyme activity was measured by the tetrapeptide substrate method. Determination of optimal pH: The pure enzyme solution was placed in different pH buffer (pH 2–pH 11), and the enzyme activity was determined by tetrapeptide-substrate method (Citric acid was used for pH 2.0–5.0, disodium hydrogen phosphate and sodium dihydrogen phosphate were used for pH 6.0–8.0, and sodium carbonate was used for pH 9.0–11.0). Determination of pH stability and temperature stability: The wild-type rAprY and mutant pure enzyme solutions were incubated at different pH or different temperatures in buffer solution at 4°C for 30 min, and the residual enzyme activity was measured. The highest enzyme activity was defined as 100%. Determination of half-life: The wild type and mutant pure enzyme solution were incubated at 55°C for 10–100 min, respectively, and the half-life (t_1/2_) of the wild type and mutant was determined. The activity of the untreated enzyme treated with high temperature was defined as 100%.

### 2.6 Site-directed mutagenesis and production of nattokinase mutants

The NNK-based site-directed mutagenesis libraries were constructed using the one-step PCR approach. The primers listed in Supplementary Table S1 were synthesized by Shanghai Sangon Biotech Co., Ltd. The plasmid pET-28a-*AprY* was used as a template for constructing mutants by site-directed mutagenesis (17). The *AprY* gene mutations were introduced via PCR using the forward primer X-up, reverse primer X-down, and TransStart FastPfu Fly PCR SuperMix (Beijing TransGen Biotech Co., Ltd., China) (34). The PCR products underwent treatment with DMT enzyme (Beijing TransGen Biotech Co., Ltd., China) before being transformed into *Escherichia coli* BL21 competent cells. The transformed cells were then spread onto an LB plate with kanamycin. The resulting colonies were sequenced by Shanghai Sangon Biotech Co., Ltd. (Shanghai, China) (34).

### 2.7 Optimization of the fermentation process of A216K production

In order to investigate the effect of different single-factor conditions on nattokinase production, the enzyme activity of A216K crude enzyme, measured by the fibrin plate method, was employed as an examination index for single-factor optimisation experiments. The optimal results of each round of optimisation were accumulated and incorporated into the subsequent round of optimisation. LB medium was used for the optimisation of enzyme production conditions, and the factors examined included fermentation time (14, 16, 18, 20, and 22 h), fermentation temperature (14, 16, 18, 20, and 22°C), bottling volume (5, 10, 15, 20, 25%), initial pH (5, 6, 7, 8, and 9), and inoculum volume (0.5, 1.0, 1.5, 2.0, and 2.5%).

### 2.8 Statistical analysis

GraphPad Prism 10.2.3 software was used forthe statistical analysis. Results are presented as the mean ± standarddeviation (SD) of the indicated number of replicates. Statistical significance was determined by a one-way ANOVA.

## 3 Results and discussions

### 3.1 Mutation strategy design based on MSC method

**Figure 1:**
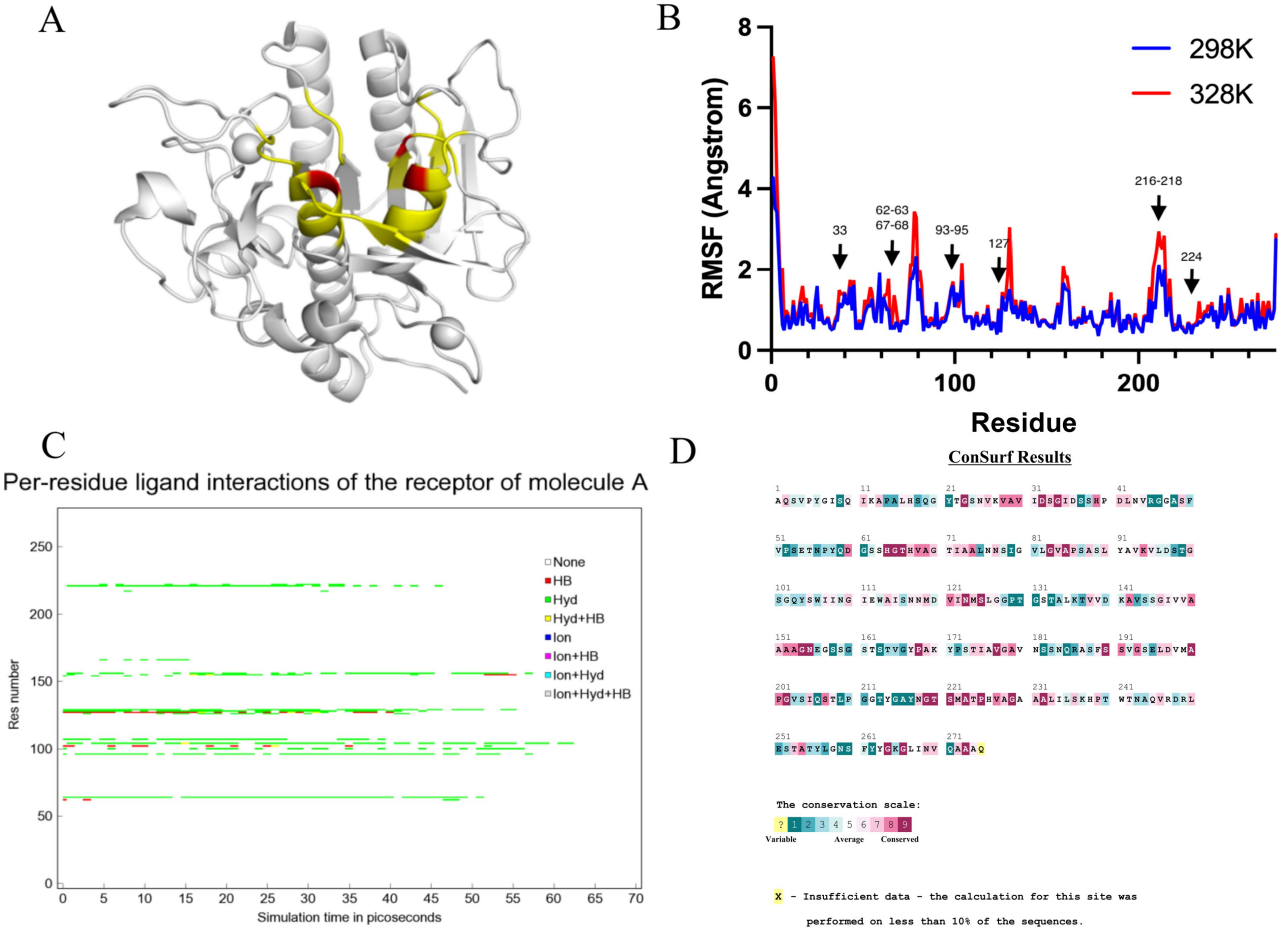
Molecular dynamics simulation at different temperatures, steered molecular dynamics simulation, and sequence conservative analysis (MSC) strategy to predict the nattokinase active centre region of nattokinase with elevated thermal stability sites. **(A)** Selection of candidate sites for AprY. The catalytic triad of AprY is labelled in red and candidate sites are indicated in yellow; **(B)** RMSF of AprY at 298 and 328 K; **(C)** Changes in substrate (sucAAPF-pNA) interaction with wild-type AprY and each mutant during SMD; **(D)** conservation prediction of AprY residues.

Amino acid residues around the active site of an enzyme have an important influence on its catalytic properties. Modifications of the enzyme active centre have been used to improve enzyme activity (20) and substrate specificity (35), etc., but the relationship between changes in the conformation of the enzyme active centre and its stability has rarely been studied. Thermostability modification studies of nattokinase have not addressed its active centre region, while active centre modifications using directed evolution tend to result in a higher workload and a high percentage of negative results. Therefore, it has become imperative to devise a rational design scheme for the stability modification of the active centre of nattokinase that reduces the intensity of screening work while improving the prediction accuracy. Molecular dynamics simulation (MD) is a powerful tool for analyzing protein structure and has been widely used to study protein function, stability, and interactions with small molecules (36). MD can predict how biomolecules interact at the molecular level, as well as the effects of mutations and ligands (37). In general, the greater the flexibility of the enzyme at high temperatures compared to room temperature, the greater the effect on the stability of the enzyme. By comparing the RMSF results at 330 K and 360 K, Yang et al. successfully enhanced the thermostability and activity of transglutaminase from *Streptomyces mobaraenesis* (38). In order to mine the more flexible sites in the active centre of the nattokinase, we first performed homology modelling of nattokinase AprY using the Alfafold online workstation and screened the sites around the catalytic triad of the active centre of AprY by PymoL software analysis (Figure 1A); Further, we performed 100 ns molecular dynamics simulations of AprY at 298 and 328 K temperatures, respectively, and compared the RMSF changes of the above candidate sites at different temperatures (Table 1; Figure 1B). The analyses showed that the RMSF of the majority of candidate sites at 328 K were larger than their counterparts at 298 K (Table 1), with 20 amino acid residues (33–34, 62–63, 66–68, 93–127, 154, 216–218, 224) having RMSF differences greater than 0.05 Å. We therefore selected the above candidate amino acid residues for subsequent studies.

**Table 1:** Potential thermostability-enhancing amino acid residue site results predicted by the MSC strategy.

Residues	RMSF 298 K (Å)	RMSF 328 K (Å)	ΔRMSF (Å)	Conservation^*^	Analysis of the interaction with substrate
30	0.726	0.749	0.023	8	
31	0.782	0.822	0.040	9	
**33**	**0.507**	**0.569**	**0.062**	**8**	
34	0.538	0.619	0.081	9	
61	1.325	1.215	−0.110	1	
**62**	**1.186**	**1.371**	**0.185**	**4**	
**63**	**1.018**	**1.395**	**0.377**	**5**	
65	0.515	0.541	0.026	9	
66	0.620	0.713	0.093	9	
**67**	**0.526**	**1.368**	**0.842**	**7**	
**68**	**0.701**	**1.032**	**0.331**	**8**	
69	0.651	0.692	0.041	8	
**93**	**0.718**	**0.787**	**0.069**	**7**	
**94**	**0.704**	**0.804**	**0.100**	**8**	
**95**	**0.734**	**0.821**	**0.087**	**6**	
96	0.918	0.981	0.063	4	Hydrophobic
125	0.441	0.652	0.211	9	
126	1.347	1.443	0.096	6	Hydrophobic
**127**	**1.046**	**1.285**	**0.239**	**6**	
152	0.579	0.599	0.020	8	
153	0.634	0.658	0.024	8	
154	0.526	0.579	0.053	9	Hydrophobic
155	0.887	0.889	0.002	9	
**216**	**1.047**	**1.240**	**0.193**	**1**	
**217**	**1.329**	**1.775**	**0.446**	**1**	
**218**	**0.746**	**0.908**	**0.162**	**7**	
219	0.471	0.515	0.044	9	
220	0.577	0.661	0.084	9	
222	0.888	0.897	0.009	7	Hydrophobic
223	0.596	0.603	0.007	9	
**224**	**0.529**	**0.590**	**0.061**	**7**	
225	0.462	0.476	0.014	9	

Positive results are indicated in bold.

* Candidate amino acid residues were assigned a score of 1 to 9 in descending order of conservatiion.

The use of molecular docking methods to identify key amino acid residues of enzymes that interact with substrates has been reported in some studies (15, 39). However, substrate binding to the active site of an enzyme is a dynamic process, and substrate entry into the active site of an enzyme may also be accompanied by transient interactions with other amino acid residues of the active site, which may also have an important effect on the catalytic properties of the enzyme. In order to further investigate possible interactions of candidate amino acid residues with substrates, we docked the tetrapeptide substrate with AprY, and performed steered dynamic simulations (SMD) of each complex. *Bacillus subtilis* protease 8h7p, a homologous protein of nattokinase, is the only protein family of *Bacillus subtilis* reported to have resolved true crystal structure after binding to a tetrapeptide substrate analogue (5, 6-dihydro-benzo [H]CINNOLIN-3-YLAMINE). Thus, our docking results combine similarity to 8h7p and binding free energy (−6.548 kJ/mol; Supplementary Figure S2). The SMD results show that L96, L126, G154 and M222 each have prolonged hydrophobic interactions with the substrate when the substrate is stretched from binding to the enzyme active centre to 15 Å from the enzyme active centre (Table 1; Figure 1C; Supplementary Figure S3). Considering the important role of the sites where the enzyme active centre interacts with the substrate for the catalytic performance of the enzyme, we subtracted the above four sites from the 20 candidate sites that had been screened.

A large body of literature has shown that the conservation of amino acid residues in enzyme proteins is closely related to their catalytic properties (40). Mutations targeting highly conserved amino acid residues often lead to enzyme inactivation, and therefore the elimination of these highly conserved amino acid residues is essential to improve prediction accuracy. Therefore, we performed a conservative analysis of the 16 candidate amino acid residues that had been screened and excluded the strictly conserved amino acid residues (those with a conservatism score = 9; Figure 1D; Table 1). Eventually, 13 candidates were selected for a targeted saturation mutation screen.

We performed site-directed mutagenesis of the above 13 candidate sites using NNK precision primers and identified sites with higher thermostability by a strategy of screening the crude enzyme solution using the fibrin plate method. To our expectation, mutations at most of the 13 candidate sites resulted in loss of activity, whereas mutants with enhanced thermostability were found at five sites, 33 (S33T), 62 (S62A), 216 (A216E, A216K, and A216R), and 218 (N218L; Table 2). Li et al. screened mutants S33T and N281L with improved thermostability using a computational thermostability prediction algorithm, while Liu and Luo et al. also identified mutants Y217K and S62A with improved thermostability using flexible region design and surface charge engineering strategies, respectively (17, 18, 21). More interestingly, these reported mutants showed a non-significant reduction or even enhancement of activity compared to the wild type, confirming the ability of our screening strategy to overcome the activity-stability trade-off in enzyme engineering studies. To further validate the reliability of our proposed strategy, we further subjected all candidate sites in the active central region of AprY to targeted saturation mutagenesis based on NNK parsimonious primers. The results showed that no activity was detected for any of the candidate sites except for P225A, which exhibited high thermostability (Table 2). We further purified P225A and determined the enzyme kinetic parameters using tetrapeptide method, and found that the specific enzyme activity of P225A was only 39.0% of that of wild-type AprY, and the k_cat_ value also decreased by 35.6%. This result confirms that our MSC strategy can solve the trade-off problem between enzyme activity and stability while improving screening accuracy.

**Table 2:** Enzyme activity and stability results of site-directed mutagenesis using parsimonious primers.^*^

Mutation	Fibrinolytic activity (%)	Residual activity after incubation at 55°C for 30 min (%)	Mutation	Fibrinolytic activity (%)	Residual activity after incubation at 55°C for 30 min (%)
Wild type	100	47 ± 12.64	126	-	-
30	-	-	127	-	-
31	-	-	152 (A152S)	7.28 ± 5.00	-
33 (S33T)	116.11 ± 15.67	77.12 ± 13.26	153	-	-
34	-	-	154	-	-
61	-	-	155	-	-
62 (S62A)	116.67 ± 7.20	58.52 ± 9.21	216 (A216E)	112.52 ± 13.63	78.51 ± 17.82
63	-	-	216 (A216K)	127.25 ± 11.28	71.83 ± 7.01
65	-	-	216 (A216R)	112.85 ± 19.21	69.33 ± 11.97
66	-	-	217 (Y217K)	45.63 ± 11.20	62.12 ± 9.68
67 (H67L)	47.32 ± 14.67	-	218 (N218L)	73.2 ± 7.37	57.84 ± 7.57
68 (V68I)	81.04 ± 17.56	-	219	-	-
69	-	-	220	-	-
93	-	-	222	-	-
94	-	-	223	-	-
95	-	-	224	-	-
96	-	-	225 (P225A)	81.04 ± 15.6	78.33 ± 13.74
125	-	-	225 (P225S)	76.56 ± 16.65	39.00 ± 17.31

* The experiments were conducted using the fibrin plate assay to ascertain the crude enzyme form of each mutant. The enzyme activity was defined as 100% for the non-thermal incubation of the wild-type AprY. A dash (−) indicates that no activity was observed or that the activity did not reach the minimum detection limit.

In summary, we selected three previously unreported mutants, A216E, A216K and A216R, with both increased activity and thermostability, from the screened positive sites for subsequent characterisation studies.

### 3.2 Purification and characterization of A216E, A216K, and A216R mutants

**Figure 2:**
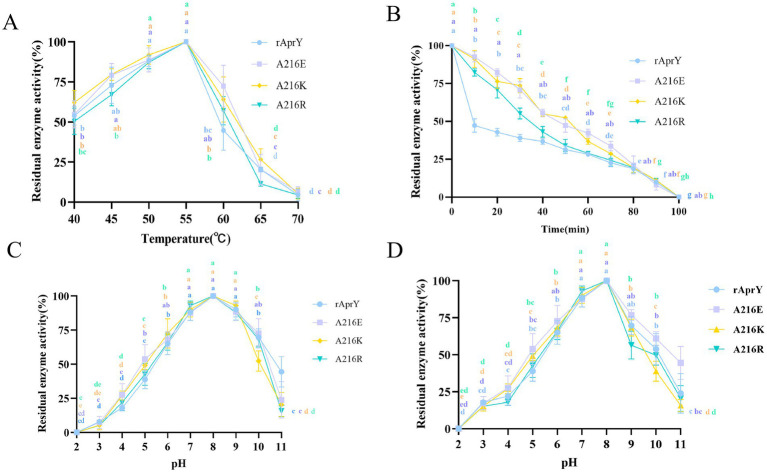
Enzymatic properties of rAprY and its mutants with single-point mutations. (A) Optimum temperature; (B) Half-life period; (C) Optimum pH; (D) pH stability.

In order to further confirm the enzymatic properties such as activity and thermostability of the selected mutants, rAprY and three mutants A216E, A216K, and A216R were purified and characterized (Supplementary Figure S4), and their enzymatic properties were detected using the tetrapeptidase substrate method, and the results are shown in Table 3 and Figure 2. A216E, A216K, and A216R mutants showed significantly increased half-life at 55°C (5.5-, 5.7-, and 3.6-fold, respectively) compared with wild type rAprY (Figures 2A,B). The surface charge of a protein is related to its optimal pH and pH stability (41). It is noteworthy that neither acidic nor basic mutations of amino acid residue 216 resulted in alterations to the pH optimum or pH stability (Figures 2C,D). However, surface electrostatic potential analysis of each mutant indicated a change in charge at the mutation site (Supplementary Figure S5). The kinetic parameters of rAprY and its mutants were determined and were presented in Table 3. Analysis of the enzyme kinetic parameters of the A216E, A216K, and A216R mutants revealed the underlying mechanisms for the increase in specific enzyme activity. The *K*_m_ value of A216K mutant decreased compared with the wild-type rAprY, indicating a better substrate affinity; In addition, the *k*_cat_ values of A216E and A216R mutants increased by 29.7 and 187.0%, respectively, indicating their superior catalytic efficiency.

**Table 3:** Comparison of the enzymatic properties of the wild-type rAprY and the single point mutants.

	Wild-type rAprY	A216E	A216K	A216R
*K*_m_ (μmol/L)	1.52 ± 0.08	2.44 ± 0.12	1.29 ± 0.04	2.27 ± 0.36
*k*_cat_ (S^−1^)	7.10 ± 0.69	9.21 ± 0.32	5.53 ± 0.16	20.38 ± 0.33
*K*_d_ (*k*_cat_/*K*_m_)	4.67	3.77	4.28	8.97
Specific activity (U/mg)	398.27 ± 35.6	428.98 ± 25.4	483.15 ± 50.6	436.40 ± 18.9
half-life (min)	9.80 ± 0.68	54.30 ± 1.2	55.93 ± 0.98	35.36 ± 0.96
Optimal pH	8	8	8	8
Optimum temperature (°C)	54	56	56	56

**Figure 3:**
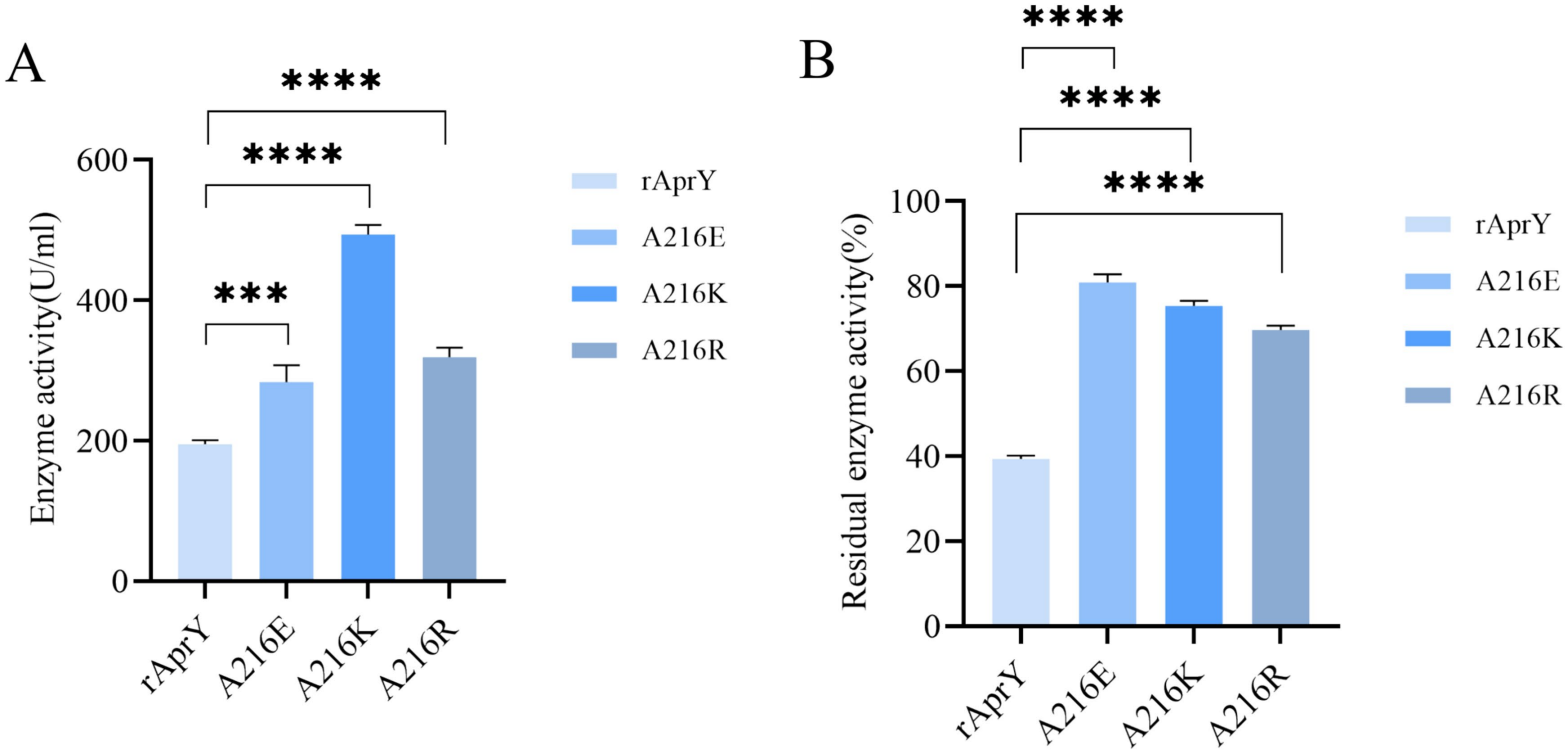
Nattokinase activity (A) and thermostability (B) measured using the fibrin plate assay. ***p < 0.001, ****p < 0.0001 were considered statistically significant vs. the rAprY group.

The fibrin plate method is affected by incubation time, plate thickness and temperature, so the measured data cannot be used to accurately reflect the activity of nattokinase, but it can be used to characterise thrombolytic activity (7). Tetrapeptide substrates, as synthetic compounds, are often used as substrates for the determination of protease activity and enzymatic properties of the *Bacillus subtilis* protease family. However, this could not accurately simulate the thrombolytic activity of nattokinase, which is one of the few proteases in the *Bacillus subtilis* protease family that can hydrolyse the clot. We further examined the activity and thermostability of pure nattokinase and its mutants by fibrin plate assay on the basis of the tetraptide substrate assay. The standard curve was made with urokinase standard, and the activity of nattokinase was calculated by measuring the diameter of transparent circle. The results of nattokinase enzyme activity are shown in Figure 3, where wild-type rAprY, A216E, A216K and A216R enzyme activities were 190.36, 282.76, 507.17, and 333.92 U/ml, respectively. The residual enzyme activities were measured by incubating each enzyme solution at 55°C for 10 min. The results showed that the residual enzyme activities of wild-type rAprY, A216E, A216K, and A216R were 40.19, 83.08, 76.58, and 70.35%, respectively. The trend of the results was basically the same as that of the tetrapeptide substrate method.

### 3.3 Understanding the mechanism for enhanced thermostability

In order to elucidate the mechanism of the enhanced thermostability of the three mutants, we performed molecular dynamics simulations at 328 K for each of the wild type AprY and its mutants. Throughout the MD process, wild-type AprY and its mutants were structurally intact, with no shedding of calcium ions bound at the two calcium ion sites. Many studies have confirmed that enzymes with enhanced thermostability show reduced enzyme displacement, stabilised structural conformation, and reduced enzyme surface area and volume contact during high-temperature molecular dynamics (42, 43). The overall structure shows that the acidic mutant with amino acid residue at position 216 (A216E) has a decreased RMSD value, while the overall hydrogen bonding with the corresponding bond energy is slightly increased, suggesting that it may be related to the elevated rigidity; whereas the basic mutants (A216K, A216R) have an increase in RMSD, Rg and SASA (Supplementary Figure S6; Table 4). Ban et, al. identified that the 1,4-α-glucan branching enzyme enhances enzyme thermostability through interaction with potassium or sodium ions in solution. Circular dichroism analysis suggests an increased α-helix proportion in the enzyme’s secondary structure as a potential mechanism for this improved thermostability; In addition, the number of hydrogen bonds within the protein structure and between it and the solvent determines its stability (44). Although the overall conformations of A216K and A216R were more active at 328 K, the number of intra-structural and inter-structural-solvent hydrogen bonds increased, and the secondary structure composition of the proteins was not significantly altered, especially the weight of the α-helix and β-sheet, which determines that the elevation of the overall flexibility of A216K and A216R does not affect their structural stability (Table 4). It is noteworthy that the potential energy of A216K and A216R is reduced, and the reduction is mainly contributed by the Coulomb potential, which rather suggests that the stability of A216K and A216R mutants is increased compared to wild type AprY (Supplementary Table S2). None of the current point mutations against nattokinase had a significant effect on the overall structural change, and the stability of the protein is not only determined by the overall structure of the protein, but also depends on the local key regions that maintain the protein conformation (17, 33). Therefore, it is necessary to conduct an in-depth study on the conformational changes in the local regions of each mutant under 328 K conditions to explore the mechanism of the enhanced thermostability of three mutants.

**Table 4:** MD simulation results of WT nattokinase (AprY) and its mutants at 328.15 K for 100 ns.

	AprY	A216K	A216R	A216E
RMSD (Å)	1.517	1.720	1.720	1.231
Rg (Å)	16.940	17.030	17.030	16.398
SASA (Å^2^)	41793.767	42503.773	42685.316	41466.513
Total energy (kJ/mol)	−230059.886	−259498.324	−259498.324	−230450.616
α-helix (%)	30.54	30.88	30.74	30.01
β-sheet (%)	21.88	20.92	20.90	22.15
Coil (%)	14.60	13.83	13.27	14.69
β-turn (%)	32.67	34.06	34.79	32.62
The number of hydrogen bonds inside the protein	194.93	198.59	198.43	197.74
Number of protein-solvent hydrogen bonds	370.24	388.09	390.79	372.15

**Figure 4:**
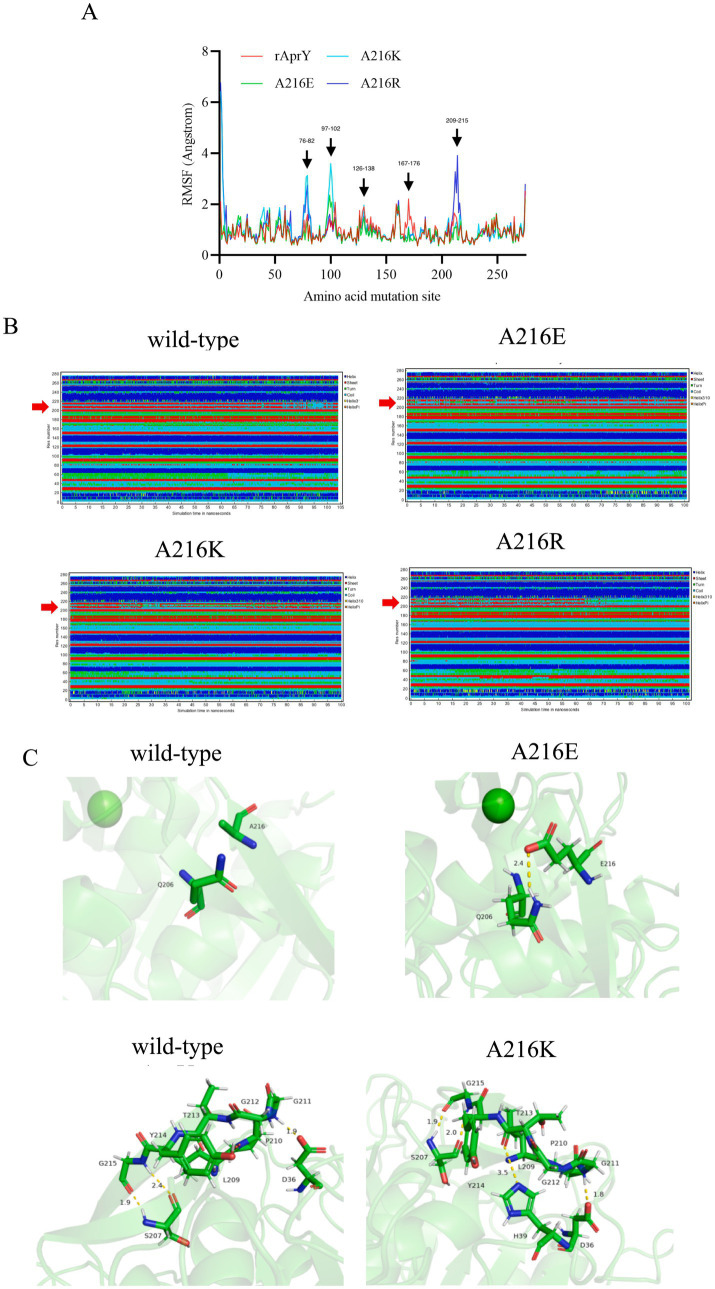
Changes in local structure of wild-type AprY and each mutant during MD at 328.15 K. (A) RMSF; (B) Changes in the secondary structure corresponding to each amino acid residue; (C) Comparison of intermolecular reactions of AprY and its mutants (all protein structures analyzed were taken from the average conformations generated during the MD procedure).

The RMSF results showed that all three mutants showed reduced RMSF in the region of residues 167–176 compared to AprY, At the same time, the flexibility of A216E and A216K in the amino acid residue region 209–215 was reduced. These altered regions were mainly found in the Loop region, and stabilisation of the Loop region has been shown to enhance protein stability (45) (Figure 4A). Positions 167–176 are around the Ca2 ion binding site, and none of the mutants showed significant changes in the secondary structure of this region (Figure 4B). Our previous study showed that the activity and thermostability-enhanced nattokinase T174V mutant had similarly reduced flexibility in the region of positions 167–176 under 328 K simulation conditions, which is consistent with the results of the present study, suggesting that conformational stabilisation of this region facilitates Ca^2+^ binding, which in turn enhances thermostability (17). Accordingly, we hypothesised that the flexible changes in the region of positions 167–176 could overcome the activity-stability trade-off problem during nattokinase modification and is a good indicator for screening and evaluation. In addition, the A216E and A216K mutants showed reduced RMSF around the mutation site and more stable secondary structures around these two mutation sites compared to AprY, indicating improved structural stability in the local region of the mutation site (Figures 4A,B). Luo et al. showed that the RMSF of the nattokinase M4 mutant with elevated stability and activity was reduced at positions 202–218 at 400 K, and at the same time demonstrated that this region has an important effect on the stability of nattokinase (18). Structural analysis using the energy nadir model of each mutant during the MD process revealed that in the A216E mutant, Glu216 formed an additional hydrogen bond with Gln206, which enhanced its local stability, while in the A216K mutant, Thr213 formed an additional hydrogen bond with His39, and what’s more, Thr213 and His39 belonged to two neighbouring loop regions, and therefore the new hydrogen bond was able to stabilise the conformation of the two loop regions, which in turn enhanced the local rigidity of the protein. In addition, we additionally found that the A216E mutant has decreased flexibility in the region of positions 126–138, and our previous study also appeared to conclude that the thermostability of nattokinase is highly correlated with the flexibility in this region. We believe that the change in flexibility in this region can overcome the activity-stability trade-off problem during nattokinase modification and is a good indicator for screening and evaluation (Figure 4C).

### 3.4 Fermentation process optimisation to enhance the yield of A216K mutants

**Figure 5:**
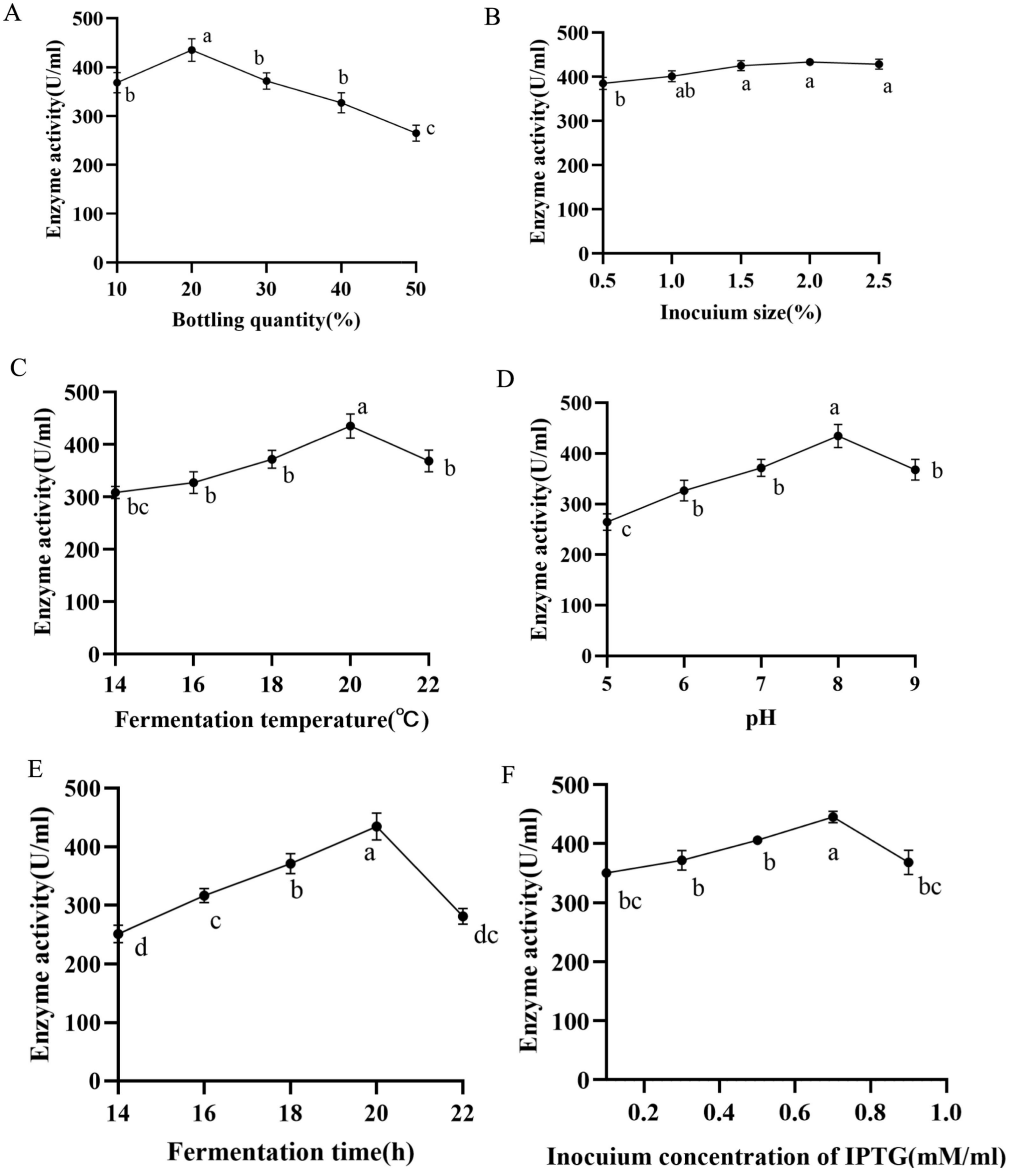
Effects of different bottling quantity (A), inocuium size (B), fermentation temperature (C), pH (D), fermentation time (E), and induction concentration of IPTG (F) on the production of nattokinase.

The heterologous expression of nattokinase in *Escherichia coli* is characterised by low expression levels and susceptibility to inclusion body formation (7). Consequently, we conducted a sequential optimisation of the fermentation conditions (bottling volume, inoculum volume, induction temperature, pH, induction time, IPTG concentration) of the A216K mutant in *Escherichia coli*. A single factor experiment was conducted to determine the optimal volume of bacterial solution to be bottled. The results indicated that a volume of 20% of the total bottling volume resulted in optimal expression, with a value of 440.55 U/ml. In light of these findings, further investigation was conducted to examine the influence of inoculum quantity and induction temperature on the expression of the A216K mutant. The results demonstrated that the impact of inoculum quantity on the expression of the A216K mutant was relatively minor in comparison to that of the induction temperature. The expression of the A216K mutant at a concentration of 449.28 and 449.64 U/ml was achieved simultaneously under the conditions of a 2% inoculum quantity and an induction temperature of 20°C. The pH of the fermentation broth had a significant impact on the metabolism and enzyme expression in *Escherichia coli* (46). The A216K mutant expression was further elevated to 453.34 U/ml when the pH was 8. The induction time was identified as a key determinant of soluble protein expression. Our single factor experiment, which varied the induction time, demonstrated that 20 h represented the optimal induction time for the A216K mutant, with an expression level of 461.14 U/ml. Induction times either above or below this optimal point resulted in a substantial decrease in expression. Finally, the impact of IPTG concentration on protein expression was investigated. The A216K mutant exhibited the highest expression at an IPTG addition of 0.7 mM/ml, reaching 467.43 U/ml (Figure 5).

## 4 Conclusion

The poor thermostability of nattokinase restricts its potential applications. There is a lack of studies targeting the modification of the active centre of nattokinase to enhance its catalytic performance. Furthermore, the direct use of directed evolution often leads to a high percentage of negative results and a significant workload. In this study, a rational design strategy combining MD-SMD-conservative analysis (MSC) was employed to reduce the screening intensity and improve the prediction accuracy of the active centre of nattokinase. This approach led to the identification of mutant A216K, which exhibited enhanced thermostability and increased activity. The new rational design strategy proposed in this study is expected to provide novel insights and methodologies for the advancement of enzyme engineering research.

## Data Availability

The original contributions presented in the study are included in the article/Supplementary material, further inquiries can be directed to the corresponding authors.
