# Supplementary material for: Modification of the active centre of nattokinase to enhance its thermostability using a strategy based on molecular dynamics simulation, steered dynamics simulation, and conservative prediction
Source: Front Nutr. 2024 Nov 14;11:1505584. doi: 10.3389/fnut.2024.1505584 (PMC11602293; doi:10.3389/fnut.2024.1505584)
Supplement: Supplementary file 1 [file Data_Sheet_1.docx]

**Support Information**

Modification of the active centre of nattokinase to enhance its thermostability using a strategy based on molecular dynamics simulation, steered dynamics simulation and conservative prediction

Yuan Li 1,†, Wenhui Zhu 1,†, Liangqi Chen 1, Xiyu Tang 1, Aixia Ma 1, Yuwei Ma 1, Tongli Li 1, Xingrui Li 2*, Ye Ma 2,*and Jinyao Li 1*

1 Xinjiang Key Laboratory of Biological Resources and Genetic Engineering, College of Life Science and Technology, Xinjiang University, Urumqi 830017, China

2 School of Pharmaceutical Sciences and Institute of Materia Medica, Xinjiang University, Urumqi 830017, China

* Correspondence: [xrli@xju.edu.cn,](mailto:xrli@xju.edu.cn,) maye@xju.edu.cn, ljyxju@xju.edu.cn

† These authors contributed equally to this work.

Table S1 Design of nattokinase mutant primers

| Primer name | Gene sequence(5’-3’) |
| --- | --- |
| V30-F | aacgtaaaagtagctNNKatcgacagcg |
| V30-R | KNNagctacttttacgttagagcctgtg |
| I31-F | gtaaaagtagctgttNNKgacagcggaa |
| I31-R | KNNaacagctacttttacgttagagcct |
| S33-F | gtagctgttatcgacNNKggaattgact |
| S33-R | KNNgtcgataacagctacttttacgtta |
| G34-F | gctgttatcgacagcNNKattgactctt |
| G34-R | KNNgctgtcgataacagctacttttacg |
| G61-F | aacccataccaggacNNKttctcacg |
| G61-R | KNNgtcctggtatgggtttgtttcagaa |
| S62-F | ccataccaggacggcNNKtctcacggta |
| S62-R | KNNgccgtcctggtatgggtttgtttca |
| S63-F | taccaggacggcagtNNKcacggtacgc |
| S63-R | KNNactgccgtcctggtatgggtttgtt |
| G65-F | gacggcagttctcacNNKacgcatgtcg |
| G65-R | KNNgtgagaactgccgtcctggtatggg |
| T66-F | ggcagttctcacggtNNKcatgtcgccg |
| T66-R | KNNaccgtgagaactgccgtcctggtat |
| H67-F | agttctcacggtacgNNKgtcgccggta |
| H67-R | KNNcgtaccgtgagaactgccgtcctgg |
| V68-F | tctcacggtacgcatNNKgccggtacga |
| V68-R | KNNatgcgtaccgtgagaactgccgtcc |
| A69-F | cacggtacgcatgtcNNKggtacgattg |
| A69-R | KNNgacatgcgtaccgtgagaactgccg |
| V93-F | gcatcattatatgcaNNKaaagtgcttg |
| V93-R | KNNtgcatataatgatgcgcttggcgct |
| K94-F | tcattatatgcagtaNNKgtgcttgatt |
| K94-R | KNNtactgcatataatgatgcgcttggc |
| V95-F | ttatatgcagtaaaaNNKcttgattcaa |
| V95-R | KNNttttactgcatataatgatgcgctt |
| V96-F | tatgcagtaaaagtgNNKgattcaacag |
| V96-R | KNNcacttttactgcatataatgatgcg |
| S125-F | gatgttatcaacatgNNKcttggcggac |
| S125-R | KNNcatgttgataacatccatattgttg |
| L126-F | gttatcaacatgagcNNKggcggacctt |
| L126-R | KNNgctcatgttgataacatccatattg |

| Primer name | Gene sequence(5’-3’) |
| --- | --- |
| G127-F | atcaacatgagccttNNKggacct |
| G127-R | KNNgacatgcgtaccgtgagaactgccg |
| A152-F | atcgtcgttgctgccNNKgccggaaacg |
| A152-R | NNKggcagcaacgacgataccgctggaa |
| A153-F | gtcgttgctgccgcaNNKaacgaag |
| A153-R | KNNtgcggcagcaacgacgataccgctg |
| G154-F | gttgctgccgcagccNNK aacgaaggtt |
| G154-R | KNNggctgcggcagcaacgacgataccg |
| N155-F | gctgccgcagccggaNNKgaaggttcat |
| N155-R | KNNtccggctgcggcagcaacgacgata |
| A216-F | ggaggcacttacggcNNKtaacggaa |
| A216-R | KNNtgcggcagcaacgacgataccgctg |
| Y217-F | ggcacttacggcgctNNKaacggaacgt |
| Y217-R | KNNagcgccgtaagtgcctccaggaagt |
| N218-F | acttacggcgcttatNNKaacgtcca |
| N218-R | KNNataagcgccgtaagtgcctccagga |
| G219-F | tacggcgcttataacNNKacgtccatgg |
| G219-R | KNNgttataagcgccgtaagtgcctcca |
| T220-F | ggcgcttataacggaNNKtccatggcga |
| T220-R | KNNtccgttataagcgccgtaagtgcct |
| M222-F | tataacggaacgtccNNKgcgactcctc |
| M222-R | KNNggacgttccgttataagcgccgtaa |
| A223-F | aacggaacgtccatgNNKactcctcacg |
| A223-R | KNNcatggacgttccgttataagcgccg |
| T224-F | ggaacgtccatggcgNNKcctcacgttg |
| T224-R | KNNcgccatggacgttccgttataagcg |
| P225-F | acgtccatggcgactNNKcacgttgccg |
| P225-R | KNNagtcgccatggacgttccgttataa |

Table S2 The contribution of the components of the potential energy in the wild-type AprY and its mutants at 328K.

|  | AprY (kJ/mol) | A216E (kJ/mol) | A216K (kJ/mol) | A216R (kJ/mol) |
| --- | --- | --- | --- | --- |
| Bond | 25024.40 | 25062.71 | 27786.27 | 27795.25 |
| Angle | 18127.86 | 18133.01 | 19465.95 | 19468.75 |
| Dihedral | 65237.07 | 63644.49 | 71300.87 | 71341.62 |
| Planarity | 509.04 | 503.62 | 499.87 | 497.77 |
| Coulomb | -369134.08 | -367987.05 | -414600.75 | -414588.82 |

A





B





C





D





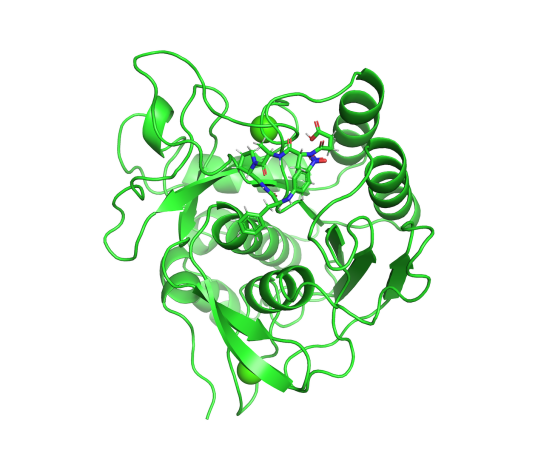
Figure S1 Ramachandran plots of AprY and its variants:(A) AprY, (B) A216E, (C) A216R, (D) A216K. Residues in the most favored regions are shown in red; Residues in additionally allowed regions are shown in yellow.

Figure S2 Results of suc-AAPF-pNA docking with Wild type AprY. The highest resolution crystal structure PDB (ID:4DWW) of nattokinase was used as the model for homology modeling. *Bacillus subtilis* protease 8h7p, a homologous protein of nattokinase, is the only protein family of *Bacillus subtilis* reported to have resolved true crystal structure after binding to a tetrapeptide substrate analogue (5, 6-dihydro-benzo [H]CINNOLIN-3-YLAMINE). Wild type AprY binding energy= -6.548 kJ/mol.


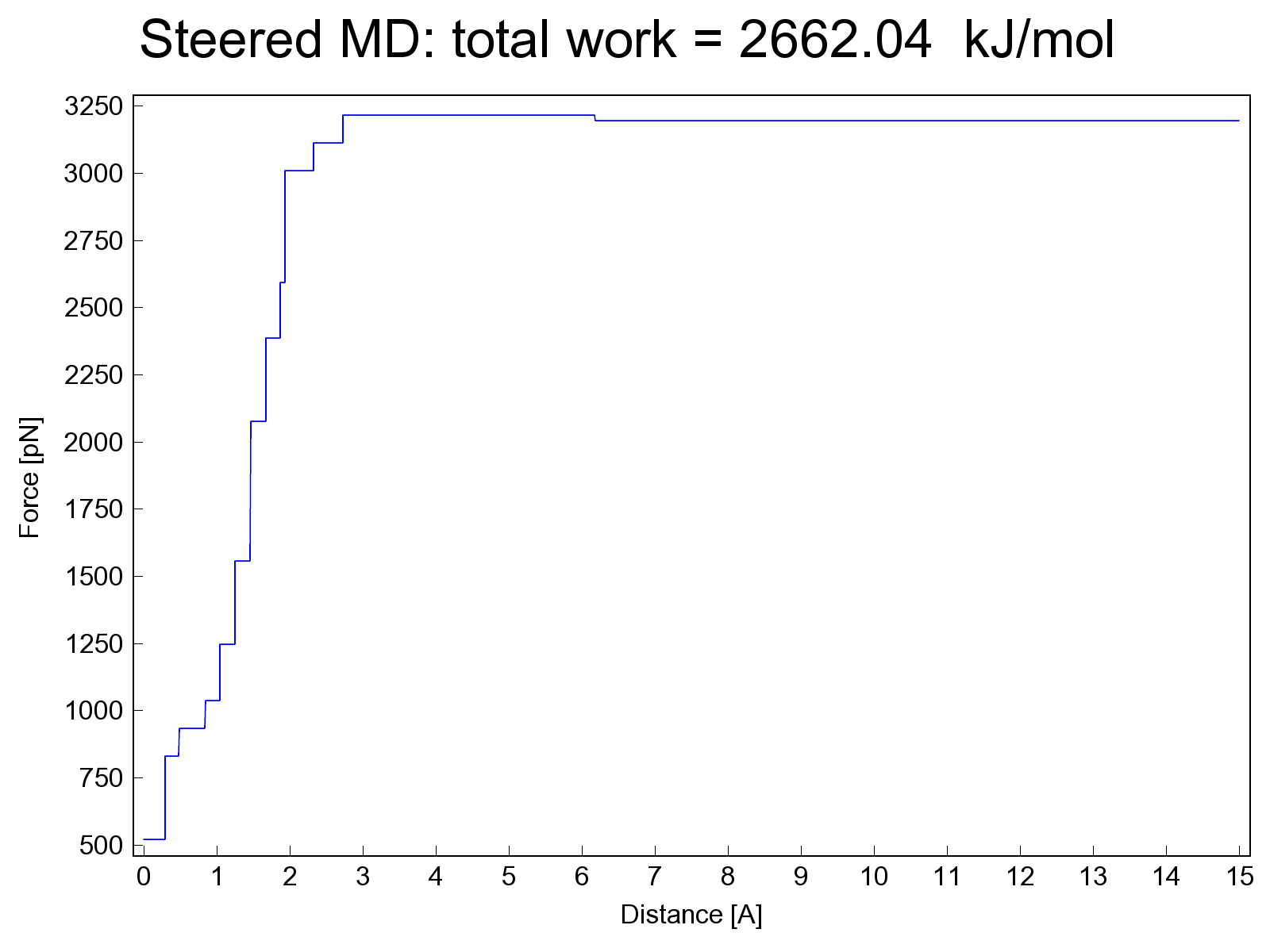


Figure S3 Changes in steered force during steered dynamics simulations.


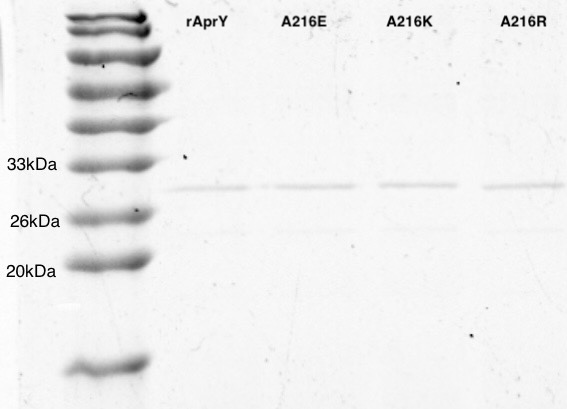


Figure S4 SDS‐PAGE of purified AprY and its variants. Lane M: Standard marker protein

with molecular weights 10–190 kDa.

Figure S5 Surface charge of wild type AprY and its variants. Amino acid residue 216 of the wild-type AprY (A), A216E (B), A216K (C) and A216R (D) is marked with a black dashed box.


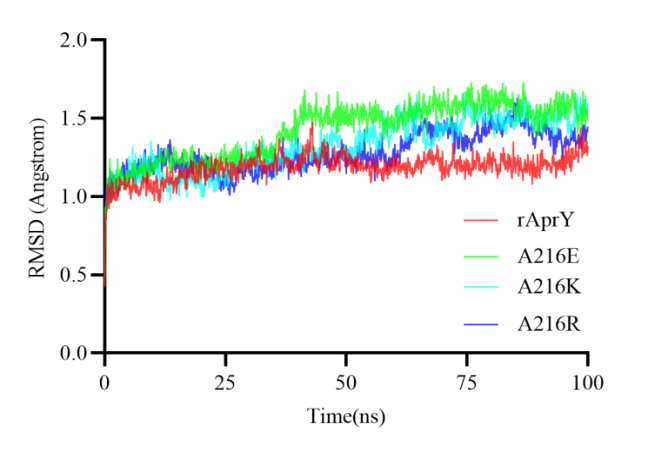

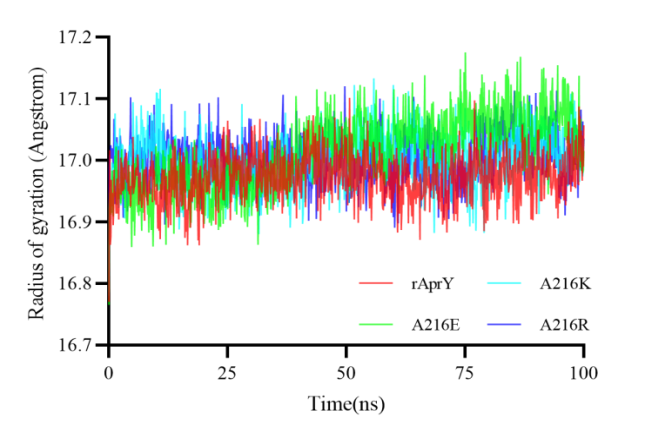

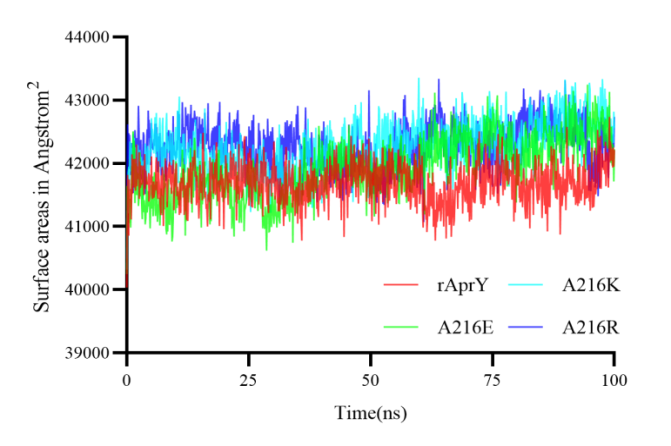

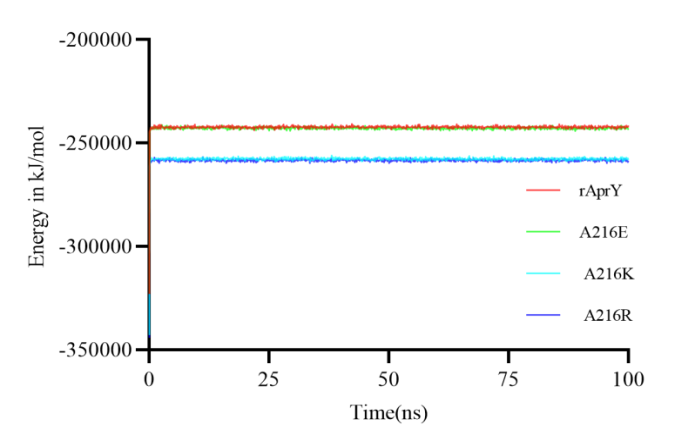


A

B

C

D

C

B

A

D


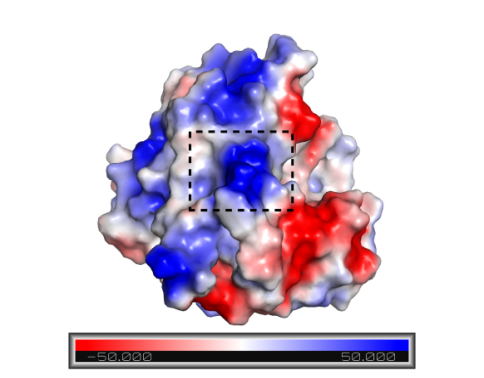

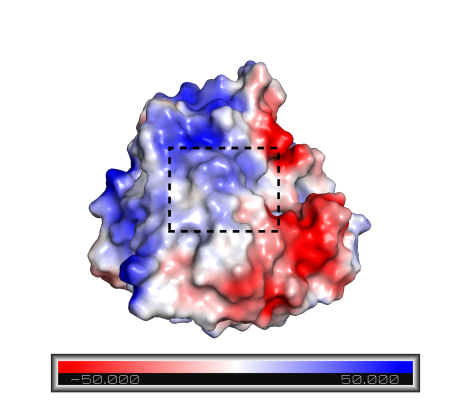

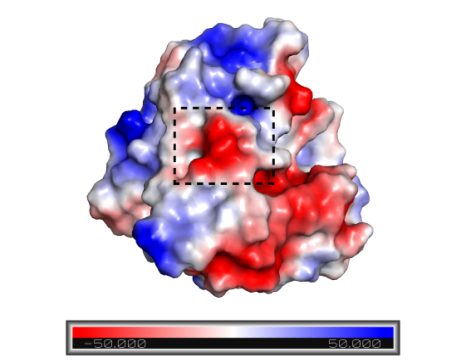

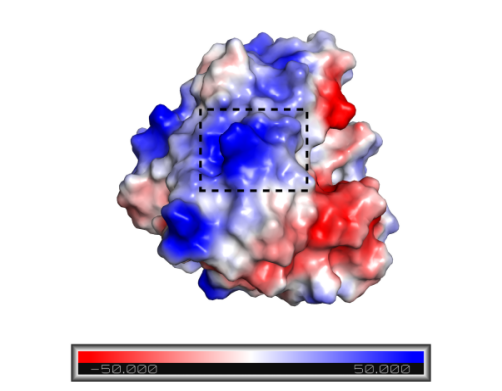


Figure S6 MD simulation results of AprY and its mutants at 328.15K for 100ns: (A)RMSD;(B) Rg;(C) SASA;(D) Total energy.
